# Tuberculosis in HIV/AIDS patients in Arad

**DOI:** 10.1186/1471-2334-13-S1-P76

**Published:** 2013-12-16

**Authors:** Mirandolina Prişcă, Dana Negru, Liliana Bran

**Affiliations:** 1 Infectious Diseases Department, Arad Emergency Clinic, Romania; 2 Public Health Department, Arad, Romania; 3 Arad Medical College, Romania

## Background

HIV and tuberculosis (TB) are so closely connected that their relationship is often described as a co-epidemic. Dual infection with HIV and TB leads to reciprocal interactions that have significant clinical impact. The approach to the treatment of tuberculosis in the HIV-infected patient is complex and must address the patient's requirement for antiretroviral therapy, potential drug reactions, and complications related to the immune reconstitution inflammatory syndrome (IRIS). Poverty and poor access to services are challenges for the successful treatment of those affected by TB and HIV.

## Methods

A case control study was set up to determine the proportion of patients with HIV/AIDS and TB co-infection among the patients attending for medical assistance, to correlate its prevalence with the Deprivation Index. Data were collected from medical records and from interviewing patients about their social and economic status.

## Results

Prevalence of tuberculosis infection is increased eight times in HIV/AIDS patients compared to the general population. TB co-infection risk is proportionally increased with deprivation and disadvantage higher scores. People with no income or low income, and/or with low level of education, and/or homeless, and/or with difficult access to health services, are statistically significantly correlated (p<0.0001) with the presence of TB co-infection. Relative risks of TB co-infection increase for people with higher disadvantage scores, and/or low income, and/or unemployed status and/or homeless (p<0.0022). Association of TB infection is correlated with B3, C2, C3 stage of HIV infection (p=0.000) compared with other cases. Of 150 patients, 14.66% presented also TB infection. The relative risk for HIV/AIDS detection in C3 stage in a TB patient is 7.2 times higher than in the absence of co-infection (p<0.0001).

## Conclusion

Lack of awareness of TB or HIV status can prevent adequate treatment. TB disease is, in fact, an AIDS-defining condition. Those living in poverty suffer from a lower quality of life and present an increased risk for the disease, including infectious diseases.

